# Reports on Cities, Towns, & Districts

**Published:** 1855-06

**Authors:** 


### SANITARY AND SOCIAL SCIENCE.

### REPORTS ON CITIES, TOWNS, & DISTRICTS.

### I.—SWANSEA (*continued*).

"WHAT IS LOCAL IS OF THE GENERAL."

When sanitary science in its infancy had to contend against opponents at every step, it was a favourite allegation that however excessive might be the mortality of any district, no evidence was forthcoming to prove it due to local preventible causes, and that it was in every case but the accident of the employments of the population, the peculiar position of the town, or other matters which were beyond control, and not remediable by the appliances of science or art. In no one thing have sanitary reformers accomplished greater results than in proving that mortality ever bears an inverse ratio to the amount of sanitary supervision and care in any given district, and in demonstrating that those accustomed to the statistics of disease, and its causes and prevention, need but an inspection of a locality to form a very close approximation to its absolute and relative immunity from disease and death. Take any town, divide it into districts, named successively 1, 2, and 3; in the first division place those streets where good pavements, free drainage, good ventilation, and where a copious and pure supply of water obtain; in the second list place those streets where these advantages are only partial; and in the third, where the gutters are neglected, the cesspits overflowing, the courts unpaved, the floors of cottages occasionally muddy from the overflow of water, where garbage lies on the unpaved streets, and where human ordure meets and offends the eye at almost every step, and the results will be uniformly the same—not, indeed, precisely in absolute numbers, but generally expressed it will be found that No. 1 has but half the mortality of No. 2, and No. 2 but half the sickness, disease, suffering and death, which prevail in the last division. In other words, by our neglect of nature's first laws of preservation, the abodes of our humbler brethren entail upon them and their offspring a fourfold mortality—clearly depending on no peculiarity of soil, of employment, occupation, of atmosphere, or food—but the penalty man pays for his fellow-man's greed.

Pursuing this sub-division of the town of Swansea, we find in it the following per centage of deaths to inhabited houses in—

| No. I. Class. | | No. II. Class. | |
|---|---|---|---|
| Adelaide Street | 26 | College Street | 50 |
| Belle Vue Street | 29 | Clarence Street | 74 |
| Castle Bailly Street | 21 | Clarence Terrace | 50 |
| Wind Street | 36 | Dillwyn Street | 35 |
| Singleton Street | 37 | Edward Street | 40 |
| Mean | 29 | Mean | 50 |

No. III. Class.

| | | | |
|---|---|---|---|
| Bethesda Street | 100 | Howell's Court | 90 |
| Croft Street | 80 | Garden Street | 80 |
| Dyvalty Street | 52 | Queen Street | 117 |
| Ebenezer Street | 84 | Tontine Street | 100 |
| James Street | 93 | | |
| John Street | 115 | | |
| Troy Street | 78 | Mean | 90 |

The same comparative result, although not in so great a ratio, follows an inquiry into the ages of those dying in various localities, the mean of the age of those living in the better localities being nearly double that of the latter class; and this element of age is one of considerable importance in determining the sanitary condition of any locality. Thus it will be found, by consulting Table I, that immense loss of duration of life, and consequent injury to the community, occur in the town of Swansea.

We therefore see from Table I, that in the best years ten years is lost to each individual born in the town of Swansea; that only half born ever attain the age of twenty years; and that more than one-third die before the age of five years. Now from this table it follows that the excess in number of deaths every year in the town is 197; that at the most moderate computation every individual loses nine years and three months of life, and every adult seven years; and supposing that the labour of each adult is worth 7*s.* 6*d.* per week, this makes a total loss of productive labour of £136 : 10 to each adult. The unnecessary sickness, at a very moderate computation, would cost £1,462 : 10; unnecessary treatments at £2 each with housing, &c. £394; and lost labour to the community of £38,220; making an annual total loss of the enormous sum of £40,000—drifting down the ocean of time, wanting a hand to snatch by sanitary regulation this mass of human life and treasure from destruction. It is true the population of the town of Swansea increases rapidly, doubling itself in a cycle of twenty-five years; but this is dependent less on its own inherent strength than on a constantly flowing tide of immigration. Thus we find that, in the ten years ending 1850, the births exceeded the deaths by 1,749. But the increase of population amounted to 5,329; it therefore follows that 3,580 persons, or 358 annually, have come from other districts to reside in the town. It is, however, satisfactory to note the progress of the past five years. Thus in

| | | |
|---|---|---|
| 1850 | there was an excess of births over deaths amounting to | 312 |
| 1851 | " " " | 139 |
| 1852 | " " " | 307 |
| 1853 | " " " | 245 |
| 1854 | " " " | 435 |
| | Total | 1438 |

Or nearly as great an excess in the past five years as in the ten years immediately preceding.

Table II illustrates the prevalence of particular classes of disease in the four quarters of the year.

TABLE I.—BOROUGH OF SWANSEA: ACTUAL RATES OF MORTALITY.

| NAME. | Date. | Proportion of Deaths to 1000 of Population. | Proportion of Deaths Annually. | Proportion of Births to Population. | Deaths from Epidemics. | Average Age of all who have Died. | Average Age of all who have died above 20 Years. | Proportion per Cent. of Deaths at each interval of Deaths to Total Number. UNDER 1 Year. | UNDER 5 Years. | UNDER 15 Years. | UNDER 20 Years. | BETWEEN 20 and 30. | BETWEEN 30 and 40. | BETWEEN 40 and 50. | BETWEEN 50 and 60. | BETWEEN 60 and 70. | BETWEEN 70 and 80. | BETWEEN 80 and 90. | Above 90. |
|---|---|---|---|---|---|---|---|---|---|---|---|---|---|---|---|---|---|---|---|
| SWANSEA TOWN Males | Mean of 5 Years | .. | .. | .. | .. | .. | .. | 21.5 | 40.1 | 48.1 | 52.0 | 8.4 | 7.6 | 8.8 | 5.5 | 7.6 | 5.8 | 3.1 | .6 |
| SWANSEA TOWN Females | | .. | .. | .. | .. | .. | .. | 16.8 | 36.8 | 44.8 | 48.2 | 7.6 | 7.8 | 7.6 | 6.1 | 7.8 | 8.0 | 4.7 | 1.7 |
| Mean | .. | 24.68 | 1 in 40 | 1 in 31 | About 1 in 200 | Yrs. Ms. 20 7 | Yrs. Ms. 53 0 | 18.7 | 38.5 | 46.5 | 50.1 | 8. | 7.7 | 8.2 | 5.8 | 7.7 | 6.9 | 3.9 | 1.2 |
| Mean | 1854 | 18.74 | 1 in 53 | 1 in 27 | .. | 26 8 | 50 4 | 20.6 | 37.9 | 46.2 | 49.5 | 12.9 | 5.6 | 7.5 | 5.2 | 7.8 | 6.9 | 3.7 | .7 |
| Average of 61 healthy districts, containing upwards of a million inhabitants | .. | 16.01 | 1 in 62½ | 1 in 35 | 1 in 449 | 37 5 | 60 0 | 16. | 27.5 | 34.4 | 38.4 | 7.8 | 5.9 | 5.6 | 6.8 | 10. | 13.3 | 10. | 2.0 |

### Table II.—BOROUGH OF SWANSEA: MEAN MORTALITY OF FIVE YEARS IN QUARTERLY PERIODS.

| CAUSES OF DEATH. | Jan. 1st to April 1st. | | | | April 1st to July 1st. | | | | July 1st to Oct. 1st. | | | | Oct. 1st to Jan. 1st. | | | | Totals. |
|---|---|---|---|---|---|---|---|---|---|---|---|---|---|---|---|---|---|
| | 0 to 15. | 15 to 60. | Above 60. | All Ages. | 0 to 15. | 15 to 60. | Above 60. | All Ages. | 0 to 15. | 15 to 60. | Above 60. | All Ages. | 0 to 15. | 15 to 60. | Above 60. | All Ages. | All Ages. |
| All causes | 87 | 53 | 42 | 182 | 88 | 59 | 31 | 179 | 64 | 58 | 29 | 151 | 83 | 49 | 23 | 155 | 667 |
| Specified causes | 85 | 52 | 42 | 179 | 86 | 58 | 31 | 175 | 62 | 58 | 29 | 149 | 81 | 48 | 22 | 151 | 654 |
| 1. Zymotic (or epidemic, endemic, and contagious diseases) | 22 | 3 | 1 | 26 | 32 | 4 | 1 | 37 | 28 | 21* | 6 | 55 | 31 | 5 | 2 | 38 | 156 |
| SPORADIC DISEASES. | | | | | | | | | | | | | | | | | |
| 2. Dropsy, cancer, and diseases of variable and uncertain seat | 0 | 2 | 2 | 4 | 0 | 2 | 2 | 4 | 0 | 2 | 0 | 2 | 0 | 1 | 2 | 3 | 13 |
| 3. Tubercular diseases | 6 | 17 | 3 | 26 | 4 | 20 | 2 | 26 | 3 | 14 | 1 | 18 | 4 | 16 | 2 | 22 | 92 |
| 4. Diseases of the brain, spinal marrow, nerves, and senses | 22 | 5 | 5 | 32 | 22 | 3 | 3 | 28 | 17 | 3 | 4 | 24 | 21 | 6 | 3 | 30 | 114 |
| 5. Diseases of the heart and blood vessels | 1 | 1 | 2 | 4 | 0 | 3 | 1 | 4 | 1 | 2 | 1 | 4 | 0 | 2 | 2 | 4 | 16 |
| 6. Diseases of the lungs and other organs of respiration | 17 | 12 | 12 | 41 | 14 | 11 | 10 | 35 | 4 | 5 | 8 | 17 | 11 | 7 | 8 | 26 | 119 |
| 7. Diseases of the liver, stomach, and other organs of digestion | 2 | 5 | 2 | 9 | 3 | 5 | 3 | 11 | 1 | 3 | 2 | 6 | 2 | 3 | 2 | 7 | 33 |
| 8. Diseases of the kidneys | 0 | 0.3 | 0.3 | 0.6 | 0 | 1 | 0 | 1 | 0 | 1 | 0 | 1 | 0 | 1 | 0 | 1 | 3.6 |
| 9. Childbirth and diseases of the uterus | 0 | 0 | 0 | 0 | 0 | 2 | 0 | 2 | 0 | 2 | 0 | 2 | 0 | 2 | 0 | 2 | 6 |
| 10. Rheumatism, diseases of the bones, joints, &c. | 1 | 0 | 0 | 1 | 0 | 1 | 0 | 1 | 0 | 1 | 0 | 1 | 0 | 1 | 0 | 1 | 4 |
| 11. Diseases of the skin, cellular tissue, &c. | 2 | 2 | 1 | 5 | 2 | 2 | 0 | 4 | 1 | 0 | 0 | 1 | 0 | 0 | 0 | 0 | 10 |
| 12. Premature births and debility | 2 | 0 | 0 | 2 | 2 | 0 | 0 | 2 | 0 | 0 | 0 | 0 | 1 | 0 | 0 | 1 | 6 |
| 13. Atrophy | 8 | 1 | 0 | 9 | 5 | 0 | 0 | 5 | 6 | 0 | 0 | 6 | 9 | 1 | 0 | 10 | 30 |
| 14. Age | 0 | 0 | 13 | 13 | 0 | 0 | 9 | 9 | 0 | 0 | 6 | 6 | 0 | 0 | 0 | 0 | 28 |
| 15. Sudden | 0 | 1 | 1 | 2 | 0 | 1 | 0 | 1 | 0 | 1 | 0 | 1 | 0 | 1 | 0 | 1 | 5 |
| 16. Violence, privation, cold, and intemperance | 2 | 3 | .. | 5 | 2 | 3 | 0 | 5 | 1 | 3 | 1 | 5 | 2 | 2 | 1 | 5 | 20 |
| Causes not specified | 2 | 1 | 0 | 3 | 2 | 1 | 1 | 4 | 2 | 0 | 0 | 2 | 2 | 1 | 1 | 4 | 13 |

* Including Cholera.

W. H. MICHAEL.

### SANITARY CONDITION OF LAMBETH SQUARE AND THE SURROUNDING DISTRICTS.

Abstract of a Report presented to the General Board of Health, by Dr. Donald Fraser, Superintending Medical Inspector. [The Report is dated Oct. 11, 1854.]

Since the prevalence of cholera in this district in 1849, improvements had taken place as to covering over ditches, and making communications into them from what were still open untrapped privies. Beyond this, a few houses in various streets which had trapped closets, remained in much the same state as in 1849. One great exception to this charge, however, was Lambeth-square, which contained 35 eight-roomed houses belonging to one owner, and occupied by a superior class of mechanics, and others of a corresponding position in life. The number of inmates was about 434, or not quite 13 to each house. In 1849 only four houses at the north-west extremity of the square had proper waterclosets; all the others had open trapped privies, draining by faulty brick drains into a 24-inch barrel drain, running down the centre of the square. In 1852 all the houses were furnished with proper closets, the old drains taken up, and 9-inch pipes laid down. To this improved drainage may be attributed the immunity of Lambeth-square from cholera in 1854. In 1849 there were six fatal cases; during 1854, up to October 8th, there was not one case. In 1850 the mortality was only six, including one case of measles. In 1851—23, including 16 zymotic cases, 4 of small-pox, 10 of scarlet fever, one of measles, and one of typhus fever. In 1852 it fell to 12; only one case of zymotic disease (small-pox) having occurred January, previously to the square being redrained. In 1853 the mortality was only six, but this included two cases of scarlet fever. During 1854 mortality was high, but there was only one zymotic case, namely, scarlatina.

Although there was an almost entire absence in 1854 of that class of disease which would propagate itself in a foul atmosphere, there was more than an average amount of those diseases which denote degeneracy of constitution, such as arise from defective nutrition, or from the children or their parents having long inhaled an impure atmosphere, containing a considerable amount of decaying animal or vegetable particles.

Further evidence of the same kind is given, as obtained from the inhabitants of other houses in the square.

There are 2,196 houses in this district, containing at the last census 18,295 individuals. The registrar, Mr. Daws, stated that the mortality had diminished, and this he ascribed to several open ditches which traversed the district in 1849, and received the contents of the privies, having been since then covered over, and many of the cesspools converted into trapped privies draining into the sewer. A striking instance of sanitary improvement, probably attributable to this circumstance, occurred in Francis-street, where, in 1849, 14 deaths were occasioned by cholera; during 1854 only one took place—a woman who had been for several days in a house at Debtford, where seven of her relatives had died of cholera, and who was taken ill a few hours after her return to Francis-street.

The mortality in all the district up to the 8th October, was from cholera, 101 deaths against 256 in the year 1840, in addition to about 16 cases of diarrhœa.

In the streets contiguous to the graveyard of Waterloo church, the mortality from all causes used to be very high; but since the closure of the graveyard, which was in a disgusting condition, the mortality has greatly diminished.

Dr. Fraser states, that there were many nuisances in this district, as slaughter-houses, manure-heaps, and piggeries.

Dr. Fraser gives a communication which he had received from Mr. Dodd, the medical officer of the district. It describes the condition of several parts of the district, and is confirmatory of the statements made by Dr. Fraser. One extract will show the unhealthy state of the district.

"There is little to complain of in the Waterloo-road, or the streets on the right hand, till you reach Granby-street, which is nearly devoted to women of the lowest class. We have much disease here, arising from their dissolute and intemperate habits. This is bounded by James-street, where we constantly have cholera, fever, and all kinds of eruptive diseases, owing, I believe, to the food purchased in the next street, the Cut or Lower Marsh. This place is infested with every description of costermongers, who sell all kinds of animal and vegetable produce, the refuse of other places, and so poison the poor in this locality. This is a market for fish of the most wretched, stinking quality. There are also many butchers who slaughter their own meat, and the cleansings, &c., pass into the common sewer, which, for want of being trapped, and being open in many places, emits abominable stinks at times, so that the people living opposite are often obliged to cover the gratings with loose straw. On the farther side of this street are numerous streets inhabited by the lowest classes, and very much overcrowded, frequently twenty and often thirty persons being in one house. These houses generally have no back premises, and all the wash is thrown out into the street. This district is partly supplied by the Vauxhall Water Company, and the water is of a poisonous character, full of dirt and animal matter; many of the water-butts are kept in such a bad state, and placed so near the privies, as to render the water unfit for drinking use."

Dr. Fraser recommends that all the privies should be made water-closets, and a better system of house drainage should be effected. There should be a market inspector appointed to prevent decomposed vegetables and tainted fish or meat being sold, and an unlimited supply of water to the whole neighbourhood. The plugs of the mains should be opened much more frequently, and the street drains washed down once a week. There should also be an intelligent and trustworthy inspector of nuisances, whose duties should be confined to the special business for which he was appointed, and who should make a daily return to the parochial authorities, or their representatives, of the places he had visited.

### MORTALITY OF BOMBAY, AND ITS CAUSES.

The total mortality of Bombay from all causes during the six years from 1848 to 1853 inclusive was 82,865. Of this number 45,730 were males, and 37,135 were females. The diseases which caused death were as follows: cholera, small-pox, measles, fever; diseases of the nervous, vascular, respiratory, alimentary, and urinary systems; of the sexual and child-bearing systems; of the locomotive and tegumentary systems; cachexy, debility, and syphilis; leprosy, tubercle, articular diseases, dropsy, accident, and violence. Of these diseases, the most fatal was fever: the total number of deaths from this cause being 33,495, or nearly one-half the total mortality. Cholera and diseases of the alimentary systems rank next: the total for the former being 13,561, or about one-sixth, and for the latter 11,234, which is nearly one-seventh of the total number of deaths. Small-pox causes a small part of the mortality of Bombay, 4687 deaths being registered, or about one-seventeenth part. Diseases of the respiratory organs, and of the nervous system, also caused a large proportion of the deaths.

The total mortality of Bombay among the different castes for the same six years was:—

| | | | |
|---|---|---|---|
| Boodhist—Jain, &c. | | Jew | 3014 |
| Brahmin | 7168 | Native Christian | |
| Lingaet | | Indo-European | 674 |
| Hindoo of other Caste | 47,077 | European | 1381 |
| Hindoo, Out-Caste | | Negro African | 556 |
| Moosulman | 18,064 | Unknown and other Castes | 79 |
| Parsee | 4802 | | |

The period of life most fatal appears to be that between the ages of 22 and 33 years: 14,126 deaths being registered, including both sexes.

The mortality at various periods of life is widely different to that which it attains in this country. Thus, in Bombay, the mortality of children under two years is greatest on the male side; from two to a period varying from seven to ten the case is reversed, and the larger mortality appears on the female side. From this period up to eighty a marked change again takes place in favour of the mortality of males; while after eighty the preponderance once more turns to the female side. These modifications are so widely opposed to what occurs in this country, that they have excited not a little surprise in our minds. To understand the matter fully, we require to have the population of Bombay, as it existed at the various periods of life during the years specified. It is more than probable, that the fluctuations of mortality vary with the fluctuations in the number of each sex constituting the community. The small proportion of small-pox is owing to the effective system of vaccination carried on in Bombay. (Compiled from statistical tables in the *Transactions of the Medical and Physical Society of Bombay*, vol. ii. New Series, 1855.)

